# Force level of small diameter nickel-titanium orthodontic wires ligated with different methods

**DOI:** 10.1186/s40510-017-0175-z

**Published:** 2017-08-01

**Authors:** Rodrigo Hitoshi Higa, José Fernando Castanha Henriques, Guilherme Janson, Murilo Matias, Karina Maria Salvatore de Freitas, Fernanda Pinelli Henriques, Manoela Fávaro Francisconi

**Affiliations:** 1 Department of Orthodontics, Bauru Dental School, University of São Paulo, Alameda Octávio Pinheiro Brisolla 9-75, Bauru, SP 17012-901 Brazil; 2 Department of Orthodontics, Ingá Dental School, Maringá, Brazil

**Keywords:** Orthodontic wires, Orthodontic brackets, Comparative study, Mechanical phenomena

## Abstract

**Background:**

The aim of this study was to compare the deflection force in conventional and thermally activated nickel-titanium (NiTi) wires in passive (Damon Q) and active (Bioquick) self-ligating brackets (SLB) and in conventional brackets (CB) tied by two different methods: elastomeric ligature (EL) and metal ligature (ML).

**Methods:**

Two wire diameters (0.014 and 0.016 in.) and 10 specimens per group were used. The specimens were assembled in a clinical simulation device and tested in an Instron Universal Testing Machine, with a load cell of 10 N. For the testing procedures, the acrylic block representative of the right maxillary central incisor was palatally moved, with readings of the force at 0.5, 1, 2, and 3 mm, at a constant speed of 2 mm/min and temperature of 36.5 °C.

**Results:**

The conventional NiTi released higher forces than the thermally activated NiTi archwires in large deflections. In general, the SLB showed lower forces, while the ML had higher forces, with both showing a similar force release behavior, constantly decreasing as the deflection decreased. The EL showed an irregular behavior. The active SLB showed smaller forces than passive, in large deflections.

**Conclusions:**

The SLB and the ML exhibit standard force patterns during unloading, while the elastomeric ligatures exhibit a randomly distributed force release behavior.

## Background

The orthodontic wires used in the alignment and leveling phase have undergone a great evolution in recent years. Nickel-titanium (NiTi) wires presented great emphasis because of their properties of superelasticity and shape memory, which make their use proper for the initial stages of orthodontic treatment [1–6]. With the development of metallurgy, NiTi wires with improved properties have been developed.

For a controlled tooth movement, light and continuous forces have been indicated [7]. In order to achieve the force levels suitable for alignment and leveling phase, it is necessary to know the force-deflection characteristics of the wires. Currently, with access to technology, it is possible to measure the forces released by the different wire types.

Several factors related to bracket/wire combination can influence the force released to the teeth, such as arch dimension, amount of deflection, ligation method, and frictional forces [8–10]. There are several ways to connect the wire to the bracket, and depending on the form chosen, the frictional force will be different. The frictional force acts as a counterforce to the forces exerted by orthodontic wires. Thus, the higher the friction, the lower the force dissipated to the teeth [11, 12].

The wire can be ligated to the bracket by means of metal ligature (ML) of different diameters, elastomeric ligature (EL), or by the specific closure system in the case of self-ligating brackets (SLB) [13]. Among the EL, the most common way to tie is the “ring” shape. Another tying option with elastomeric ligatures is the “figure 8” shape, which promotes greater pressure of the wire in the slot, increasing friction [14–17]. EL properties include light continuous force, consistent long-lasting seating archwire, resistance to water sorption, and shape memory [13]. Furthermore, they can be applied quickly, are comfortable for the patient, and have a variety of colors. However, the EL allows greater microbial accumulation on the surface of the teeth adjacent to the bracket, compared to the other ligation types, besides the fact that the archwires may not completely seat during torquing or rotational corrections, and binding may occur with sliding mechanics [13, 18–20]. Few studies have evaluated the influence of the ligation type in the force exerted by the wire on the tooth [6, 11, 12].

The use of SLB has become common in recent years. From the patients’ perspective, these brackets are more comfortable and easier to clean due to the absence of elastic or metal ligatures. Many studies have been published evaluating the frictional force produced by SLB [21–23], since the manufacturers have claimed that in these accessories there is a lower resistance force to sliding, decreasing treatment time. Although friction is not the only factor that determines treatment efficiency, it has been associated with the forces dissipated by the archwires. The different SLB designs, active or passive, can show a different behavior in relation to the friction properties. Passive brackets have shown lower friction than active brackets [24–27].

Due to the influence of the ligation methods in the force exerted on the teeth and to the extensive variation of them in the market, further studies become essential to evaluate the behavior of each wire/bracket combination. This way, the aim of the study was to evaluate the forces exerted by conventional and thermally activated NiTi wires in different ligation types, in SLB and conventional brackets (CB).

## Methods

### Material

#### Experimental groups

Three sets of brackets were selected for this study: Damon Q passive self-ligating (Ormco, Orange, California), Bioquick active self-ligating (Forestadent, Pforzheim, Germany), and Morelli conventional (Dental Morelli, São Paulo, Brazil). All brackets had a nominal 0.022-in. slot size. Two different NiTi wires were tested: conventional and thermally activated (Dental Morelli, São Paulo, Brazil), with 0.014- and 0.016-in. diameters (Table 1).

**Table 1:** Experimental groups

Wire	Diameter	Bracket and ligation type
Conventional NiTi	0.014-in.	Damon Q—passive self-ligating
		Bioquick—active self-ligating
Thermally activated NiTi	0.016-in.	Morelli—elastomeric ligature (EL)
		Morelli—metal ligature (ML)

The wires were ligated to the CB by means of “ring” shaped elastomeric ligature (RSEL) and metal ligature (ML). The wires, brackets and ligatures used belonged to the same batch, so that there were no influences in the results. The standard ISO 15841, which recommends six specimens of each sample, was used. However, to minimize the chance of any technical error and increase reliability of the results, a number of 10 specimens were chosen for each group.

For the elastomeric ligatures, tying a needle holder was used, and after insertion of the elastic, a 3-min waiting period before the tests was determined, to enable initial relaxation of the material, as recommended in other studies [28, 29]. For ML, the ligature was initially tightened with a needle holder around the wings of the bracket, and then loosened by one turn to allow free movement of the archwire.

### Methods

#### Deflection test

The evaluation tests of the force released through deflection of the orthodontic wire were performed in a clinical simulation device representing the maxillary teeth, extending from the right second premolar to the left second premolar [10, 30, 31].

**Fig. 1:**
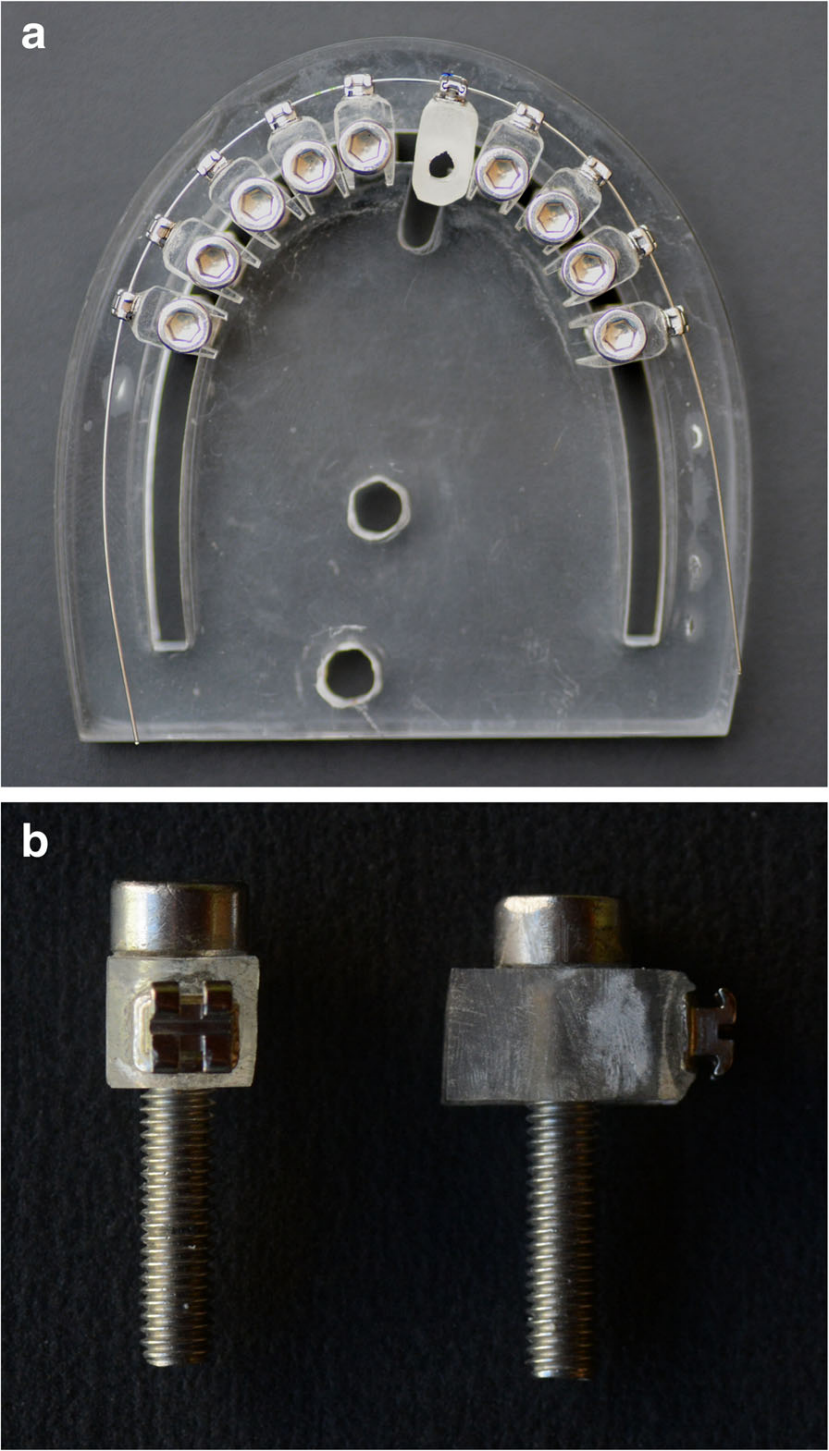
**a** The clinical simulation device used in this study. **b** Block representative of the teeth attached to the screw

Figure 1 shows the clinical simulation device that was used in this study. This device was composed of an acrylic resin plate with parabolic shape where blocks which represent the maxillary teeth were affixed. The parabola shape was determined by the wire to be tested, reducing the risk of generating diverse forces beyond the deflection applied in this study.

The blocks that represent the teeth were affixed to the acrylic plate respecting a standard distance of 6 mm between brackets [32], corresponding to the average distance between slots considering the bracket size and the average size of dental crowns mesiodistally, since the force/deflection relation is dependent, among other things, on this distance [10]. Brackets were bonded with cyanoacrylate adhesive (Super Bonder, Loctite) on acrylic blocks. These blocks were fixed by means of threaded screws to the bottom of the acrylic resin plate.

**Fig. 2:**
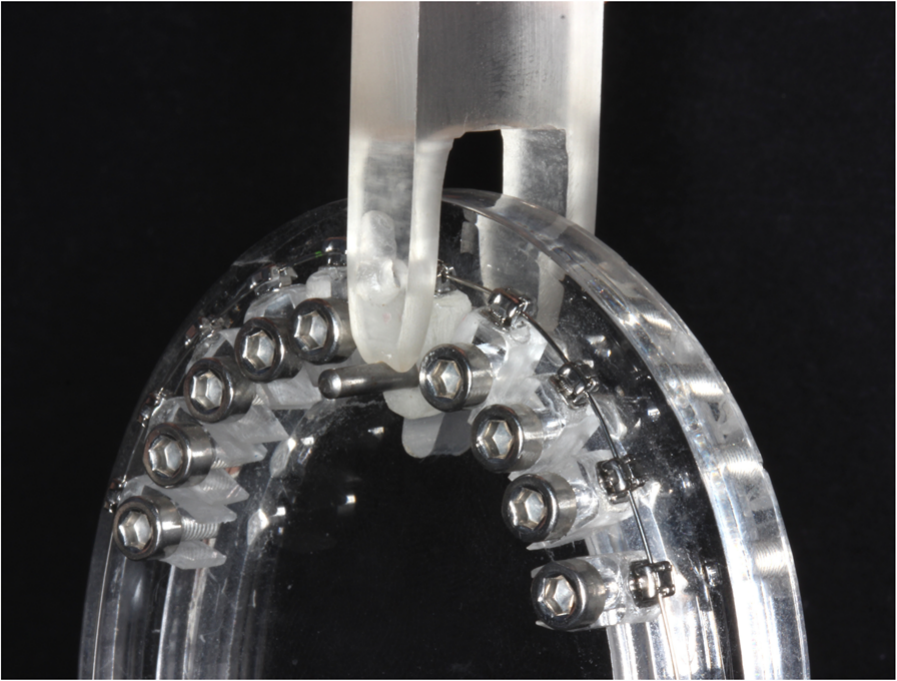
Tip of the universal testing machine moving bucco-palatally the acrylic block

The tests were performed on the block corresponding to the right maxillary central incisor. This block was not screwed, enabling its bucco-palatal movement. It received a perforation, in which a metal cylinder was placed, allowing its activation. The tip of the activation attached to the testing machine had rounded cut to fit the metal cylinder (Fig. 2). The speed of the testing machine was 2 mm/min.

**Fig. 3:**
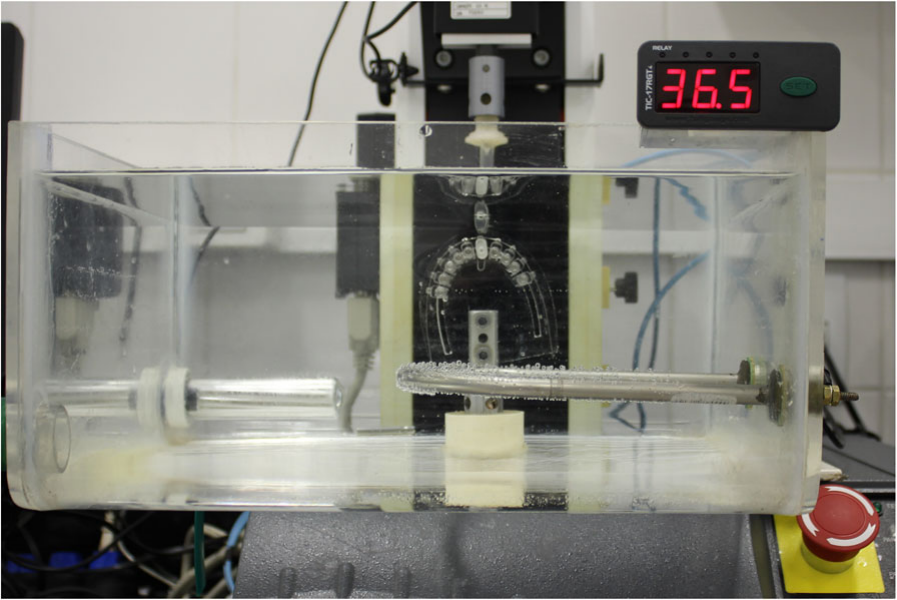
Acrylic container filled with water containing temperature control system, where the tests were conducted

To evaluate the wire deflection, an Instron 3342 universal testing machine (Norwood, MA, USA) with load cell of 10 N (1 kgf) was used. Very high load cells have no accuracy befitting with the forces dissipated by orthodontic treatment. To maintain a constant temperature of 36.5 °C in order to get closer to the reality of the oral environment, the tests were done in an acrylic container filled with water, where the temperature was controlled by a submersible electric resistance connected to a digital thermostat (TIC 17RGTi/9 model, Full Gauge Controls, Canoas/RS, Brazil) previously scheduled to stay in the desired temperature range (Fig. 3).

Before each test, load cell calibration was achieved by Bluehill Lite software (v.2.25, 2005). Assessments of wire deflection in unloading were performed beginning in 3.1 mm, and from this point, generated values could be measured in 3, 2, 1, and 0.5 mm. The deflection of the wire attached to the bracket corresponds clinically to the beginning of treatment, when the teeth are poorly positioned and the wire is forced into the slots of the accessories. Depending on the degree of crowding, teeth will experience more or less force so proper alignment occurs.

The elastic deflection test was chosen because it is clinically closest to the orthodontists’ interests, because that is what they do when adapting a wire to the patient’s teeth. Although engineers work with parameters like elastic modulus and yield value, the orthodontist is more concerned with knowing the force released in relation to the amount of deflection.

### Statistical analysis

The Kolmogorov Smirnov test was used to evaluate the normal distribution of the variables, indicating that the parametric statistical tests could be applied.

Descriptive statistics were calculated for each archwire-bracket combination.

Three- and one-way ANOVA and Tukey tests were used to compare different wires, diameters, and brackets.

All statistical analyses were performed with Statistica software (Statistica for Windows—Release 7.0 - Copyright Statsoft, Inc. Tulsa, Okla), at the *p* < 0.05 level of significance.

## Results

Table 2 (end of the manuscript) represents the results of the three-way ANOVA, considering the different archwire type, ligation system, and diameter of the archwire, in the evaluated deflections. It was found that there was influence of the different combinations in the greater deflections, but not in the smaller deflections.

**Table 2:** Three-way ANOVA for comparison of wire, bracket, and interaction wire/bracket in the different deflections

	DF	0.5 mm			1.0 mm			2.0 mm			3 mm		
		MS	*F*	*p*	MS	*F*	*p*	MS	*F*	*p*	MS	*F*	*p*
Wire alloy	1	181.55	2.260	0.135	2768	4.525	0.035	341,643	4647.44	0.000	307,140	3296.7	0.000*
Bracket	3	5380.77	66.983	0.000	20,336	33.243	0.000	25,447	346.17	0.000	70,766	759.6	0.000*
Diameter	1	30.14	0.375	0.541	45,841	74.936	0.000	315,513	4291.99	0.000	630,812	6770.7	0.000*
Interaction wire alloy/bracket	3	17.45	0.217	0.884	1345	2.198	0.091	561	7.63	0.000	4579	49.1	0.000*
Interaction wire alloy/diameter	1	6.87	0.085	0.770	365	0.597	0.441	9868	134.23	0.000	21,448	230.2	0.000*
Interaction bracket/diameter	3	351.73	4.379	0.006	1744	2.850	0.040	8242	112.11	0.000	18,182	195.2	0.000*
Interaction wire alloy/bracket/diameter	3	111.75	1.391	0.248	3318	5.423	0.001	4248	57.79	0.000	7855	84.3	0.000*

*Statistically significant at *p <* 0.05

Regarding the archwire types, the mean values of the conventional NiTi archwires were greater than thermally activated NiTi ones, but statistically significant differences were found only in 1, 2, and 3 mm of deflection. The same situation was found for the archwire diameter, indicating that the wire diameter of 0.016 in. releases greater forces than the 0.014 in.

Tables 3, 4, 5, and 6 show the means, standard deviations, and comparison of the forces in the different ligation systems by one-way ANOVA, at different amounts of deflection.

**Table 3:** Mean (in cN) and standard deviation (SD) of 0.014-in. conventional NiTi wires in the bracket systems (*n =* 10)

Elastic deflection	Damon	Bioquick	EL	ML	*p*
	Mean (S.D.)	Mean (S.D.)	Mean (S.D.)	Mean (S.D.)	
0.5 mm	24.45 (3.84) ^BC^	22.57 (6.74) ^B^	3.36 (1.64) ^A^	33.36 (12.80) ^C^	0.000*
1 mm	70.06 (8.19) ^A^	54.62 (6.12) ^A^	69.52 (41.57) ^A^	118.26 (25.26) ^B^	0.000*
2 mm	196.92 (7.79) ^B^	167.14 (6.30) ^A^	190.65 (6.36) ^B^	214.84 (7.43) ^C^	0.000*
3 mm	235.42 (8.89) ^B^	224.33 (5.62) ^A^	277.70 (9.84) ^C^	274.84 (6.47) ^C^	0.000*

*Statistically significant at *p <* 0.05

**Table 4:** Mean (in cN) and standard deviation (SD) of 0.014-in. thermally activated NiTi wires, in the bracket systems (*n =* 10)

Elastic deflection	Damon	Bioquick	EL	ML	*p*
	Mean (S.D.)	Mean (S.D.)	Mean (S.D.)	Mean (S.D.)	
0.5 mm	23.84 (2.53) ^B^	29.74 (6.99) ^B^	6.93 (7.48) ^A^	33.41 (14.70) ^B^	0.000*
1 mm	63.61 (5.83) ^A^	53.71 (8.81) ^A^	64.39 (13.46) ^A^	85.39 (13.89) ^B^	0.000*
2 mm	114.03 (6.86) ^A^	111.27 (6.96) ^A^	108.28 (6.34) ^A^	129.12 (5.66) ^B^	0.000*
3 mm	168.95 (8.00) ^A^	171.90 (7.78) ^A^	212.77 (8.41) ^C^	200.79 (8.53) ^B^	0.000*

*Statistically significant at *p <* 0.05

**Table 5:** Mean (in cN) and standard deviation (SD) of 0.016-in. conventional NiTi wires, in the bracket systems (*n =* 10)

Elastic deflection	Damon	Bioquick	EL	ML	*p*
	Mean (S.D.)	Mean (S.D.)	Mean (S.D.)	Mean (S.D.)	
0.5 mm	28.96 (5.15) ^B^	29.78 (10.64) ^B^	6.67 (7.22) ^A^	23.45 (13.48) ^B^	0.000*
1 mm	95.44 (8.66)	98.08 (10.47)	112.11 (43.07)	130.15 (55.70)	0.1359
2 mm	299.36 (11.30) ^B^	259.90 (8.18) ^A^	306.96 (9.90) ^B^	321,40 (15.76) ^C^	0.000*
3 mm	389.28 (9.16) ^B^	352.69 (7.65) ^A^	441.23 (13.10) ^D^	424,02 (5.62) ^C^	0.000*

*Statistically significant at *p <* 0.05

**Table 6:** Mean (in cN) and standard deviation (SD) of 0.016-in. thermally activated NiTi wires, in the bracket systems (*n =* 10)

Elastic deflection	Damon	Bioquick	EL	ML	*p*
	Mean (S.D.)	Mean (S.D.)	Mean (S.D.)	Mean (S.D.)	
0.5 mm	32.66 (3.10) ^B^	27.47 (5.32) ^B^	4.28 (3.56) ^A^	29.04 (16.92) ^B^	0.000*
1 mm	94.34 (6.65) ^A^	81.00 (6.67) ^A^	126.50 (41.57) ^B^	140.05 (25.26) ^B^	0.000*
2 mm	212.53 (8.01) ^B^	180.62 (4.84) ^A^	211.61 (6.19) ^B^	219.73 (10.89) ^B^	0.000*
3 mm	295.28 (12.84) ^B^	270.62 (4.88) ^A^	374.67 (15.06) ^D^	322.26 (13.23) ^C^	0.000*

*Statistically significant at *p <* 0.05

In general, for the smallest amount of deflection (0.5 mm), there was a trend, in any diameter and type of wire tested, that the force exerted by EL were much smaller than those of other ligation types.

In 1 mm of deflection, the SLB, along with the EL, showed smaller forces, while the ML showed a trend to present greater forces.

For 2 mm of deflection, the active SLB tended to have smaller forces compared to other systems. The ML showed higher forces in most tests in this deflection, while the Damon and the EL showed intermediate forces in relation to the others. Only for the 0.014 thermally activated NiTi different results were observed, with the ML releasing higher forces and the other ligation methods showing significantly similar forces among them.

In 3 mm of deflection, there was a trend for the SLB to show smaller forces, especially the active system showing smaller forces in most tests. The ML showed intermediate forces and the EL showed the highest forces for this deflection.

## Discussion

Comparing the force of the conventional NiTi wire with the thermally activated Niti, a statistically significant difference only in the two largest deflections (2 and 3 mm) was observed. The highest forces of the conventional NiTi wire are in agreement with other studies that also found similar results when comparing the two types of wire [7, 33–35]. The values found in this study, however, suggest that in small deflections there is no difference in the force exerted by these two wires in the diameters tested.

Based on these considerations, they should have different use according to the biomechanical need. In low friction mechanics, the thermally activated NiTi wires are more suitable in the alignment stage, due to their lower forces and superelastic properties compared to the conventional NiTi. However, in conventional mechanics, when the friction promoted by the ligation system is greater, these wires may be unable to overcome this resistance. Several studies have mentioned friction as one of the factors that dissipate the forces in orthodontic treatment [12, 22, 33, 36]. These studies show that low friction results in higher loads.

Behavior of the forces released was significantly variable depending on the different ligation types. In 0.5 mm of deflection, it was observed that the EL promoted very low forces in all tests (Tables 3 to 4). This result probably occurred because the force exerted by the NiTi wires was hardly enough to overcome the friction generated by the ligatures. The EL pressures the wire inside the bracket slot, increasing the friction. In applying this concept in clinical practice, force values released by this type of ligation probably would not promote tooth movement.

However, in 3 mm of deflection, which was the highest tested, the CB showed higher forces than the SLB. This occurred because the SLB does not press the smaller diameter wires inside the slot walls. However, in CB, even these wires are pressed by the elastomeric or metal ties, promoting greater deflection of the wire, which in turn results in higher levels of force. Previous studies that also compared the forces in different ligation systems corroborate with the fact that the SLB release smaller forces than CB, when smaller diameters are tested [29, 37].

The ML produced greater forces in most tests. Even so, its force release behavior was similar to the SLB, where the forces constantly decreased as the deflection decreased. It is possible that the ML behaves as an active self-ligating bracket, with the difference that it allows less freedom of the wire within the slot, compared to other SLBs. On the other hand, the force release behavior of the EL is very different from the other ligation types, releasing very high forces in large deflections and very low forces in small deflections.

In turn, the self-ligating systems show low force release rate at higher deflections, but they also release forces in small deflections, in agreement with the concept of light and continuous forces.

This concept of light and continuous forces is important because the force released for orthodontic movement is more biologically favorable, without damaging the surrounding tissues. In addition, the force is released since wire placement and remains until the new appointment, promoting constant orthodontic movement. In this sense, leveling and alignment will be more efficient.

In addition, the ligatures may change the force released due to loss of elasticity of the material, with time. A previous study found that the force released by “relaxed” elastomeric ligatures was higher than the new [12]. This probably occurs due to loss of friction with relaxation of the elastomer. However, further studies are necessary to evaluate the force after a certain period of performance of elastomeric ligatures.

Thus, it is hard to predict the amount of force released by the wire when it is connected to the bracket by means of EL. A study that examined the effect of ligation on the load-deflection characteristics of NiTi wires concluded that the EL act as a restraint on superelastic wires [11]. Therefore, the results of this study suggest that with ML and self-ligating system, predictability of the released force is greater than with the EL. The SLB has the advantage of releasing lighter forces.

When comparing the two self-ligating bracket systems, the passive (Damon Q) presented higher forces than the active system (Bioquick) in larger deflections, except for the 0.014-in. thermally activated NiTi wire. This may indicate that in situations where there is great force release, the difference between the systems appears. These situations can be related to archwires of large diameter, large deflections (larger crowding) and alloys with small superelasticity and resilience.

This force difference between self-ligating brackets can be justified by the smaller frictional force promoted by this system, demonstrated by several studies [21–23]. This is in agreement with the concept that the smaller the friction, the higher the forces [15, 38]. The results of this study suggest that in situations where there is greater force release, friction tends to exert greater interference.

In applying this concept to clinical practice, friction might influence the force in the initial stage of leveling and alignment, when crowding is severe, or in the final stage, when using a larger diameter wire. Another study compared the friction among different brackets and smaller friction was found for the Damon passive system only when larger diameter wires were used [23].

## Conclusions

- ➢ Conventional NiTi wire showed higher forces than thermally activated NiTi, in large deflections.
- ➢ The sets of low friction (self-ligating and conventional brackets tied with ML) showed more standardized forces than conventional brackets with elastomeric ligature. Metal ligature promotes greater magnitude of forces than SLB.
- ➢ The active self-ligating showed smaller forces than the passive system in large deflections.

## Abbreviations

CB: Conventional brackets
EL: Elastomeric ligature
ML: Metal ligature
NiTi: Nickel-titanium
SLB: Self-ligating brackets
